# Group B *Streptococcus* Serotypes in Pregnant Women From the Western Cape Region of South Africa

**DOI:** 10.3389/fpubh.2018.00356

**Published:** 2018-12-04

**Authors:** Charlene W. J. Africa, Eveline Kaambo

**Affiliations:** ^1^Maternal Endogenous Infections Studies (MEnIS) Research Laboratories, Department of Medical Biosciences, University of the Western Cape, Bellville, South Africa; ^2^Department of Biochemistry and Microbiology, University of Namibia Medical School, Windhoek, Namibia

**Keywords:** group B *Streptococcus*, colonization and serotypes, pregnancy outcomes, antibiotic sensitivity, South Africa

## Abstract

**Background:** Maternal colonization of Group B streptococci (GBS) during pregnancy is an important risk factor for neonatal morbidity and mortality. The aim of this study was to determine the prevalence and serotype distribution of GBS isolated from a cohort of pregnant women in the Western Cape, South Africa.

**Methods:** Two ano-vaginal swabs were collected from 301 women at 28–37 weeks of gestation. Participants were recruited from four different antenatal clinics in the Western Cape, South Africa. GBS were detected by culture and PCR and serotypes confirmed by latex agglutination tests. Antibiotic sensitivity was performed using disc diffusion.

**Results:** The GBS colonization rate was 16.6%. Serotype distribution revealed serotype V as the predominant serotype (66.67%) followed by serotype III (21.05%). Serotypes Ia, II, IV, and IX constituted 1.75% each and 3 GBS isolates were non-typeable. Serotype V demonstrated resistance to most of the antibiotics tested, while serotype III demonstrated better susceptibility, except for tetracycline. No significant differences were observed for GBS colonization or serotype distribution according to HIV status.

**Conclusion:** Predominating serotypes differed from those previously reported from other regions in South Africa. Global surveillance of **s**erotype distribution plays an important role in informing vaccine development and antibiotic prophylaxis.

## Introduction

Group B *Streptococci* (GBS) or *Streptococcus agalactiae*, may exist as part of the normal microbiota in the female genital tract and anal areas of healthy adults, with the gastrointestinal tract serving as the natural reservoir and source of vaginal colonization (1).

Maternal GBS colonization may result in pregnancy-associated conditions including urinary tract infection, bacteremia, chorioamnionitis, postpartum endometritis, preterm labor (PTL), preterm rupture of membranes (PROM) and perinatal transmission of the organism (1). Intrauterine infection has been associated with the ability of GBS to ascend from the lower genital tract and colonize the upper genital tract (2).

GBS infection in the newborn remains the foremost cause of neonatal morbidity and mortality globally, despite a recent decline in occurrence (3) with the highest incidence of invasive disease occurring in low-middle income regions such as in Southern Africa (4). It presents as early-onset disease (EOD) within 0–6 days of birth, or late-onset disease (LOD) after 7–90 days (5). Maternal colonization has been described as a prerequisite for EOD and a risk factor for LOD. Transmission from mother to infant is thought to take place vertically through aspiration of infected amniotic fluid or colonization of the newborn during passage through the birth canal (5).

Intrapartum colonization is strongly associated with early onset GBS sepsis, resulting in ~4% of reported fatalities and serious morbidities including sepsis, pneumonia, meningitis, illness, and death in infants, along with long-term disabilities (4, 6). Other well-established risk factors for early-onset invasive GBS in newborns include rupture of membranes for more than 18 h before delivery, fever in the mother during labor, preterm delivery and a history of GBS disease in previous infants.

The etiology of LOD is not as well-understood as EOD and may involve community or nosocomial acquisition (7). However, there is evidence that in several infants with LOD, the GBS causing the infection share identical serotypes as the GBS isolated from their mothers, suggesting a maternal source (7).

Intrapartum antibiotics are known to reduce EOD in many countries (5), but in underdeveloped countries, particularly in Africa where the prevalence rates are the highest, resources are limited and hinder the implementation thereof. However, the true burden of GBS colonization in many countries is not known, and the current existing information shows great variation in the colonization rates and serotype distribution in different parts of the world, thus hampering the production of a suitable vaccine.

For the above reasons, the Centres for Disease Control and Prevention (8) recommended that all pregnant women at 35–37 weeks gestation should be screened for GBS colonization using vaginal-rectal specimens in order to decrease the morbidity and mortality of GBS-associated neonatal disease. An understanding of the global variations of GBS colonization and serotype prevalence may be achieved through the study of more regions across continents, thus providing the information needed to inform the development of suitable vaccines. To this end, the present study focused on a cohort of women in the Western Cape, South Africa, with the aim of establishing their GBS colonization rates and predominant serotypes.

## Materials and Methods

### Study Population

This study included 301 women at 28–37 weeks of gestation, recruited from four antenatal clinics stationed in Mowbray, Mitchells Plain, Guguletu, and Khayelitsha in the Western Cape, South Africa. The participants were from lower socioeconomic groups, representative of two different ethnic groups, namely, black, and mixed-race groups. Patient details were captured in a brief questionnaire to which a sample number was assigned to ensure anonymity.

Excluded from the study were women who reported bacterial infections for which antimicrobials were administered within 2 weeks prior to recruitment. Also excluded were those with any reported condition for which clinical vaginal sampling may be contraindicated.

### Ethical Considerations

The content and objectives of the study were explained to the participants and their consent obtained by signature on the appropriate consent forms. Ethics approval for this study was obtained from the human ethics committee of the University of the Western Cape (UWC). The study complied with the declaration of Helsinki (9).

### Sample Collection

A vaginal and anorectal swab were collected from each patient by trained nurses, using 2 separate sterile cotton-tipped swabs. Vaginal swabs were collected by inserting the swab about 3 cm into the vaginal wall and rotating the swab circumferentially. Anorectal swabs were collected by swabbing through the anal sphincter. Swabs were transported to the laboratory in carefully labeled liquid Amies with charcoal medium (Sterilin, 18114CST, Italy) and processed within 4 h of collection.

### Sample Processing

#### Culture

Samples were inoculated in the order of first streaking onto enriched blood supplemented CNAgar (colistin nalidixic agar, Becton Dickinson, 63954, Bio-Rad Laboratories) followed by inoculation in enriched broth (Todd-Hewitt Broth) and selective broth (Todd–Hewitt broth supplemented with gentamicin: 8 μg per milliliter and nalidixic acid: 15 μg per milliliter). The agar and broths were then incubated at 37°C for 24 h (h) in order to ensure optimal recovery of GBS. Media showing no growth after 24 h were incubated for a further 24 h before being declared as negative for growth. Culture purity was confirmed by subculture onto 5% sheep blood agar and microscopy using Kopeloff's modification of the Gram stain (10).

Confirmation of GBS was achieved using catalase, Lansfield's grouping (DRO585A, Oxoid, Basingstoke), esculin hydrolysis, and CAMP (Christie, Atkins and Munch-Peterson) tests.

#### PCR

DNA extraction was based on a procedure described by Sambrook et al. (11) and the extracts separated in 0.8% (w/v) agarose gels. Primers designed for the PCR amplification of the universal bacterial and GBS-specific 16S rRNA genes are listed in Table 1. A standard 50 μl PCR reaction solution contained 1 × PCR amplification buffer (10 × buffer contained 200 mM Tris pH 7.6, 100 mM KCL, 100 mM (NH_4_)_2_ SO_4_, 20 mM MgSO_4_, 1% (w/v) Triton X-100), 0.2 mM each of dATP, dCTP, dGTP, and dTTP, 0.5 μM of each primer, 1U of *Taq* DNA polymerase, and 10 ng template DNA.

**Table 1:** Oligonucleotide specific primers used in this study for the amplification of the universal bacterial and GBS specific 16S rRNA genes.

**Primers**	**Nucleotide sequences (5′-3′)**	**Position (16S rRNA gene)**	**Annealing temp**.	**Specificity**	**References**
E9F (forward)	GAGTTTGATCCTGGCTCAG	9–27	50°C	Universal	(12)
U1510R (reverse)	GGTTACCTTGTTACGACTT	1,510–1,492	50°C	Universal	(13)
Sag 40 (forward)	CGCTGAGGTTTGTGTTTACA	40–61	60°C	Bacteria	(14)
Sag 445 (reverse)	CACTCCTACCAACGTTCTTC	445–465	60°C	Bacteria	

After the initial heating step of 94°C for 4 min, cycles totalling 30 were run under the following conditions: denaturing at 94°C for 30 s, primer annealing at 50°C for 30 s and extension @ 72°C for 1 min 30 s and final extension at 72°C for 10 min. The universal bacterial 16S rRNA gene primer pair of E9F (12) and U1510R (13) was used to amplify the nearly complete 16S rRNA gene. Amplified products were separated on 0.8% agarose gels using electrophoresis in 0.5 × TBE buffer. The products were stained with ethidium bromide and visualized under UV, using a transilluminator.

GBS specific primers (Sag 40 and Sag 445) were used for PCR amplification under the following conditions: A pre–PCR step at 94°C for 2 min, initial denaturation at 94°C for 45 s followed by 35 cycles of three temperature cycles involving denaturation at 95°C for 30 s, annealing at 60°C for 1 min, extension at 72°C for 2 min, followed by a final extension at 72°C for 10 min. The presence of each PCR product was then verified using electrophoresis in 1% agarose gel and stained with ethidium bromide. GBS template DNA was used as a positive control and no target DNA was added to the negative control.

### Serotyping

GBS serotyping of the capsular polysaccharide antigens I-IX was achieved using a latex agglutination typing kit (Hemolytic *Streptococcus* Group B typing kit, Statens Serum Institut, Denmark). A colony of GBS was suspended and incubated overnight in 5 ml of THB (Todd-Hewitt broth). Approximately 10 μl of the bacterial suspension was added to the latex suspension on the circle card and mixed by gentle shaking for a few seconds. Serotype designation was determined by a strong agglutination reaction within 5–10 s. Any agglutination after 30 s was not considered positive. If no designation could be made, the isolate was deemed non-typeable.

### Antimicrobial Susceptibility

Antimicrobial susceptibility was performed using the Kirby-Bauer disk diffusion method and results interpreted according to the Clinical Laboratory Standards Institute guidelines (15). Pure colonies from each individual isolate were suspended in 5 ml of saline. The inoculum was adjusted to the turbidity of a 0.5 McFarland standard and fresh culture suspensions (100 μl) were evenly spread on Mueller-Hinton agar plates containing 5% sheep blood. After aseptically placing the antibiotic discs on the surface of the spread plates, the plates were anaerobically incubated at 37°C overnight. The following eight antibiotic discs were used: Penicillin G (PG) 1 Unit; Clindamycin (CD) 2 μg; Gentamicin (GM) 10 μg; Fusidic Acid (FC) 10 μg; Erythromycin (E) 5 μg; Trimethoprim (TM) 1.25 μg; Sulphamethoxazole (SMX) 25 μg; Tetracycline (T) 10 μg. Post incubation, sensitivity was determined by measuring the zones of inhibition from the edge of the disk to the edge of the inhibition zone using a ruler. Cultures showing no zones of inhibition were interpreted as resistant.

### Statistical Analysis

Data were analyzed using the Statistical Package for the Social Sciences (SPSS) for windows version 19.0. The Chi square test was used to determine the associations between two categorical variables, but when the number in a cell was <5, Fisher exact test was applied. A *p* < 0.05 determined significance.

## Results

### Detection of GBS

Lancefield grouping confirmed that 57 GBS isolates were obtained from the agar and broth cultures of 50/301 (16.6%) mothers. Comparison of agar and broth cultures showed no difference in isolation of GBS, while all of the isolates which were GBS culture-positive, were also positive by PCR. There was no significant difference between GBS isolated from the vagina (26/57) and the rectum (23/57) in this study. Twenty—three samples with negative vaginal cultures were positive by their corresponding anorectal samples, while GBS was isolated from 8 mothers by both vaginal and rectal samples.

Universal bacterial primers E9F and U1510R showed that the DNA was suitable for PCR amplification and aided in determining the amount of metagenomic DNA to be used (5 ng) in all PCR experiments.

The PCR product amplified with GBS-specific forward and reverse primers (Sag 40 and Sag 445) and viewed on a 1% agarose gel, yielded the expected band size of 405 bp.

### Study Population and GBS Colonization

Although mothers from Mowbray and Khayelitsha showed similar rates of GBS colonization (36 and 32% respectively), significant differences in colonization rates were noted when Mitchells Plain (24%) and Guguletu (8%) mothers were included in the comparison (Table 2).

**Table 2:** Study population and GBS colonization.

**Variables/Categories**	**Frequency (%) *n* = 301**	**Mothers with GBS (%) *n* = 50**	**Significance**
**Location of the MOU**			*p* < 0.001
Mowbray	90 (29.9%)	18 (36.0%)	
Khayelitsha	74 (24.6%)	16 (32.0%)	
Mitchells Plain	86 (28.6%)	12 (24.0%)	
Guguletu	51 (16.9%)	4 (8.0%)	
**Age groups (years)**			*p* = 0.613
17–25	81 (27%)	15 (30%)	
26–30	107 (36%)	17 (34%)	
31–35	72 (24%)	10 (20%)	
36–40	26 (8.8%)	5 (10%)	
≥41	7 (2.3%)	2 (4.0%)	
Missing	8 (2.6%)	1 (2.0%)	
**Marital status**			*p* = 1.000
Married	134 (44.5%)	21 (42%)	
Unmarried	160 (53.2%)	26 (52%)	
Missing	7 (2.33%)	3 (6%)	
**Education**			*p* = 0.499
Primary school (G1–G7)	14 (4.7%)	2 (4.0%)	
High school(G8–12)	274 (91.0%)	48 (96.0%)	
University	10 (3.3%)	0 (0%)	
Missing	3 (1.0%)	0	
**Race**			*p* = 1.000
Black (African)	149 (49.5%)	16 (32%)	
Colored (mixed race)	41 (13.6%)	1 (2.0%)	
White	0	0	
Missing	111 (36%)	33 (66.6%)	
**Employment**			*p* = 0.925
Yes	118 (39.2%)	19 (38%)	
No	180 (59.8%)	31 (62%)	
Missing	3 (1%)	
**Personal hygiene**			*p* = 0.950
Bath	72 (24.1%)	13 (26.0%)	
Hand wash	207 (68.7%)	34 (68.0%)	
Shower	18 (6.0%)	2 (4.0%)	
Douching	1 (0.3%)	1 (2.0%)	
Missing	3 (0.9%)	
**Smoking**			*p* = 0.446
Yes	41 (13.6%)	9 (18.0%)	
No	260 (86.4%)	41 (82.0%)	
**Alcohol**			
Yes	29 (9.6%)	4 (8%)	*p* = 0.798
No	272 (90.4%)	46 (92%)	
**HIV status**			*p* = 0.440
Positive	74 (24.6%)	15 (30%)	
Negative	201 (66.8%)	31 (62%)	
Missing	26 (8.6%)	4 (8.0%)	

The mean age of the study population was 28.8 (±5.2) years and their ages ranged between 17 and 42 years (Table 2). GBS colonization was highest amongst mothers in the 26–30 year age group (24%).

Most women (53.2%) reported their marital status as unmarried and 44.52% reported being married. No response to this question was received from the remaining 2.33% (Table 2). There was no significant difference between the two groups for GBS colonization with 52% of unmarried mothers being GBS positive compared with 42% unmarried mothers.

The majority of participants indicated that they had completed high school (91%) with <5% having primary school education and only 3.3% having had tertiary education (Table 2). Three (1%) failed to reveal their educational background but were able to read and write. The higher rate of GBS colonization amongst the high school group (96%) may be attributed to them constituting the majority of participants in the study.

The sample group comprised of mostly black (African) and mixed race (Colored) mothers, the majority of whom (59.8%) were unemployed (Table 2). A large number (36%) refused to be categorized into a particular race group and 1% did not respond to the question on employment. While it would appear that GBS occurred most frequently amongst the African group (32%) compared with the Colored group (2%), many of both groups were assigned to the “missing” group (66.6%) for the reason mentioned above, thus complicating the interpretation of this data.

Hand washing of the body using a basin was the most frequently reported means of hygiene practice (68.7%), followed by bathing (24.1%), and shower (6.0%). Only 1 mother (0.3%) reported douching (Table 2). GBS prevalence was highest amongst those who reported hand washing (68%), followed by bathing (26%).

Smoking and consumption of alcohol while pregnant were reported by 13.6 and 9.6% respectively, (Table 2) with those reporting neither smoking (41%) nor drinking (46%) testing negative for GBS.

HIV positivity (HIV+) was reported by 24.6% of the mothers, with 66.8% being HIV-negative (HIV–) and 8.6% either ignorant of their status or unwilling to disclose it. GBS colonization was higher in the HIV- mothers (62%) than in the HIV+ mothers (30%), (Table 2) but this difference was not statistically significant (*p* > 0.05).

**Figure 1:**
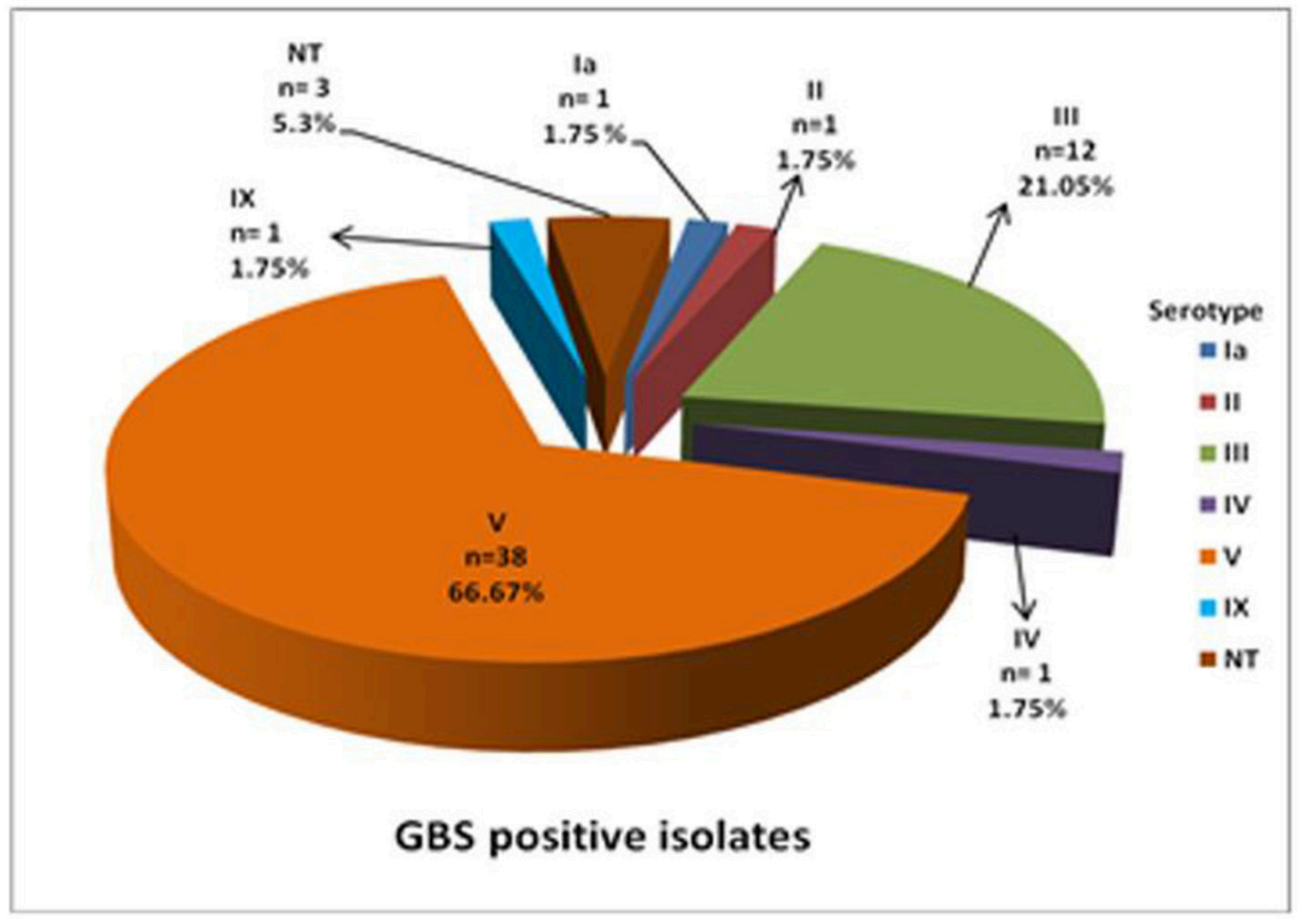
Distribution of capsular antigen types among GBS culture positive isolates from pregnant women in the Western Cape.

**Figure 2:**
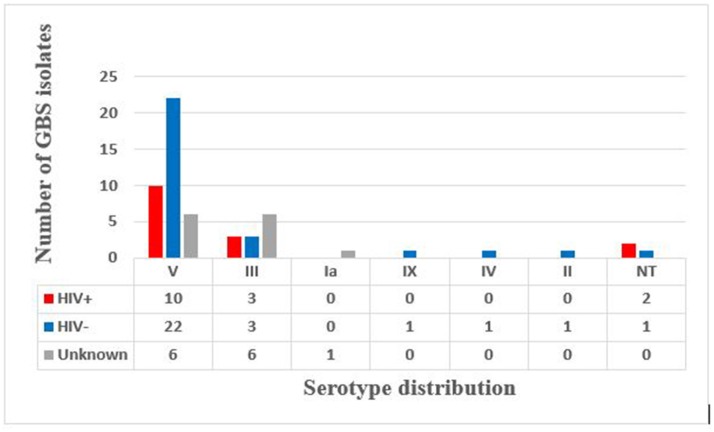
Serotype distribution according to reported HIV status.

Latex agglutination identified six different serotypes (Figure 1). Serotype V predominated (66.6%) with 17.5 and 38.5% isolated from HIV+ and HIV– mothers, respectively, and the HIV status of the remaining 10% not reported (Figure 2). Serotype III followed (21.05%), with no difference in prevalence between HIV+ and HIV– mothers (Figure 2). Only 1 (1.75%) isolate belonged to each of serotypes Ia, II, IV, and IX in our study (Figure 1), with the HIV status of the mother with serotype Ia not disclosed and serotypes II, IV, and IX isolated from HIV– mothers only (Figure 2). No significant difference in serotype prevalence was observed between HIV+ and HIV– mothers (*p* > 0.05). One woman (HIV–) harbored more than 1 serotype of GBS, namely serotypes IV and V. No strains belonging to serotypes Ib, VI, VII, and VIII were found in any of the isolates in this study. Serotyping was not possible in 3 cases (5.3%), and were thus defined as non-typeable (NT). In eight cases where GBS was cultured from both rectal as well as vaginal samples, serotyping resulted in identical findings for all of the paired samples, except for three. The remaining three women were colonized with two different serotype combinations, namely, serotypes III/IX, serotypes IV/V, and serotypes III/V from the vaginal and anorectal swabs, respectively.

### Antibiotic Susceptibility

**Figure 3:**
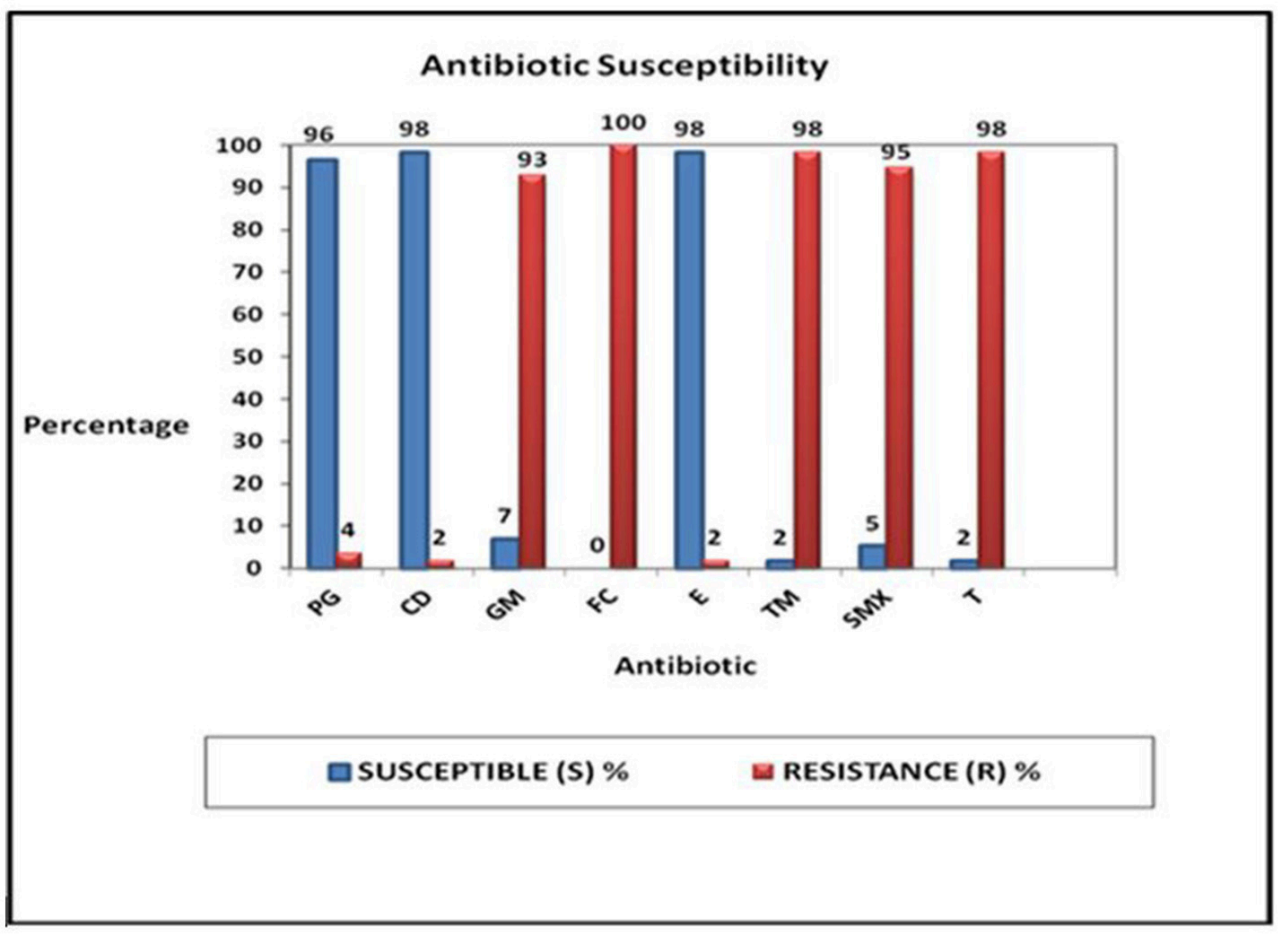
Proportions of the responses of the 57 GBS culture positive isolates tested against eight different antibiotics: penicillin G (PG), clindamycin (CD), gentamicin (GM), fusidic acid (FC), erythromycin (E), trimethroprin (TM), sulphamethoxazole (SMX), and tetracycline (T).

Fifty-six of the fifty-seven GBS isolates (98%) were susceptible to clindamycin and erythromycin and 96% (55/57) were susceptible to penicillin G. Only 2% (1/57) showed susceptibility to tetracycline (T) and trimethoprim (TM) (Figure 3). Total resistance (100%) to fusidic acid was demonstrated and 98% (56/57) were resistant to tetracycline and trimethoprim (Figure 3). Ninety-five percent of the isolates (54/57) were resistant to sulphamethoxazole, and 93% were resistant to gentamicin (Figure 3).

Examination of the susceptibility profiles of the GBS serotypes are summarized in Table 3. GBS serotypes Ia, II, IX, and the three non-typeable strains showed susceptibility to clindamycin, erythromycin, and penicillin G only. Serotype III was susceptible to clindamycin, erythromycin, penicillin G, and sulphamethoxazole. Serotype IV was susceptible to clindamycin, erythromycin, penicillin G, sulphamethoxazole, and trimethoprim. GBS serotype V showed susceptibility to penicillin, erythromycin, clindamycin with resistance to tetracycline, SMX, and TM.

**Table 3:** Antibiotic susceptibility profiles of GBS serotypes using disc diffusion.

**Antibiotic (**μ**g/disc)**												
**No (%) of susceptible isolates by serotype**	**Clindamycin (CD) 2** μ**g**			**Erythromycin (E) 5** μ**g**			**Fusidic acid (FC) 10** μ**g**			**Gentamicin (GM) 10** μ**g**		
	**Susceptible (%)**	**Resistant (%)**	**Total**	**Susceptible (%)**	**Resistant (%)**	**Total**	**Susceptible (%)**	**Resistant (%)**	**Total**	**Susceptible (%)**	**Resistant (%)**	**Total**
Ia [*N* = 1]	1 (1.75%)		1 (1.75%)	1 (1.75%)	–	1 (1.75%)	–	1 (1.75%)	1 (1.75%)	–	1 (1.75%)	1 (1.75%)
II [*N* = 1]	1 (1.75%)	–	1 (1.75%)	1 (1.75%)	–	1 (1.75%)	–	1 (1.75%)	1 (1.75%)	–	1 (1.75%)	1 (1.75%)
III [*N* = 12]	12 (21.05%)	–	12 (21.05%)	12 (21.05%)	–		–	12 (21.05%)	12 (21.05%)	–	12 (21.05%)	12 (21.05%)
IV [*N* = 1]	1 (1.75%)	–	1 (1.75%)	1 (1.75%)	–	1 (1.75%)	–	1 (1.75%)	1 (1.75%)	–	1 (1.75%)	1 (1.75%)
V [N = 38]	37 (64.91%)	1 (2.7%)	38 (66.67%)	37 (64.91%)	1 (1.75%)	38 (66.67%)	–	38 (66.67%)	38 (66.67%)	4 (7.0%)	34 (59.6%)	38 (66.67%)
IX [*N* = 1]	1 (1.75%)	–	1 (1.75%)	1 (1.75%)	–	1 (1.75%)	–	1 (1.75%)	1 (1.75%)	–	1 (1.75%)	1 (1.75%)
NT [*N* = 3]	3 (5.26%)	–	3 (5.26%)	3 (5.26%)	–	3 (5.26%)	–	3 (5.26%)	3 (5.26%)	–	3 (5.26%)	3 (5.26%)
Total (Frequency %)	56 (98.2%)	1 (1.8%)	57 (100.0%)	56 (98.2%)	1 (1.8%)	57 (100.0%)	–	57 (100.0%)	57 (100.0%)	4 (7.0%)	53 (93.0%)	57 (100%)
**Antibiotic (**μ**g/disc)**												
**No (%) of susceptible isolates by serotype**	**Penicillin G (PG) 1 unit**			**Tetracycline (T) 10** μ**g**			**Trimethroprin (TM) 1.25** μ**g**			**Sulphamethoxazole (SMX) 25** μ**g**		
	**Susceptible (%)**	**Resistant (%)**	**Total**	**Susceptible (%)**	**Resistant (%)**	**Total**	**Susceptible (%)**	**Resistant (%)**	**Total**	**Susceptible (%)**	**Resistant (%)**	**Total**
Ia [*N* = 1]	1 (1.75%)	–	1 (1.75%)	–	1 (1.75%)	1 (1.75%)	–	1 (1.75%)	1 (1.75%)	–	1 (1.85%)	1 (1.75%)
II [*N* = 1]	1 (1.75%)	–	1 (1.75%)	–	1 (1.75%)	1 (1.75%)	–	1 (1.75%)	1 (1.75%)	–	1 (1.85%)	1 (1.75%)
III [*N* = 12]	12 (21.05%)	–	12 (21.05%)	–	12 (21.05%)	12 (21.05%)	–	12 (21.05%)	12 (21.05%)	22 (38.6%)	10 (17.54%)	32 (56.14%)
IV [*N* = 1]	1 (1.75%)	–	1 (1.75%)	–	1 (1.75%)	1 (1.75%)	1 (1.75%)	–	1 (1.75%)	1 (1.75%)	–	1 (1.75%)
V [*N* = 38]	36 (63.16%)	2 (3.5%)	38 (66.67%)	1 (1.75%)	37 (64.91%)	38 (66.67%)	–	38 (66.67%)	38 (66.67%)	–	38 (66.67%)	38 (66.67%)
IX [*N* = 1]	1 (1.75%)	–	1 (1.75%)	–	1 (1.75%)	1 (1.75%)	–	1 (1.75%)	1 (1.75%)	–	1 (1.85%)	1 (1.75%)
NT [*N* = 3]	3 (5.26%)	–	3 (5.26%)	–	3 (5.26%)	3 (5.26%)	–	3 (5.26%)	3 (5.26%)	–	3 (5.26%)	3 (5.26%)
Total Frequency (%)	55 (96.5%)	2 (3.5%)	57 (100.0%)	1 (1.8%)	56 (98.2%)	57 (100.0%)	1 (1.8%)	56 (98.2%)	57 (100.0%)	3 (5.26%)	54 (94.7%)	57 (100.0%)

*NT, non-typeable*.

## Discussion

A report of invasive GBS disease in South Africa between 2004 and 2008 revealed an overall rate of neonatal GBS disease in the Western Cape as 0.53–0.67/1,000 live births (16). Neonatal GBS infection may be prevented by identifying and treating pregnant women who carry GBS or who are at highest risk of transmitting the bacteria to newborns. One recommended approach for preventing the transmission of GBS from mothers to neonates is to screen pregnant women by culture of combined vaginal and rectal regions at 35–37 weeks of gestation and to treat empirically those with positive cultures or risk factors for disease transmission. Collection of samples during the later stages of pregnancy is more representative of GBS colonization during labor.

In the present study, we followed the CDC guidelines (8) for sample collection and culture and found an overall GBS prevalence of 16.6%, compared with prevalence rates of 25, 30, and 30.9% reported in similar studies from South Africa (17–19). These differences may be attributed to several factors including ethnicity and socio-economic standards. To our knowledge, this is the first reported study of GBS serotype antimicrobial susceptibility from the Western Cape region. The aforementioned South African studies were conducted in areas inhabited by different Black (African) tribal groups and although our study reported Black participants as constituting the majority of our study group, many of the participants (36%) refused to be categorized by race, thereby introducing a bias. Historically, the Western Cape was home to the indigenous people of the region (Khoisan) and along with slaves brought from the East by European settlers, this region is predominantly inhabited by people of mixed race who previously, under *Apartheid* were classified as “Colored” (a term considered by many to be derogatory) and now fall under the generic “Black” category, which includes all race groups other than White. Because we were ethically bound to respect their refusal to be assigned to any particular race group against their will, we recorded them in the “missing” data, regardless of their appearance.

Low socio-economic status may be associated with the level of education, access to preventive health care and poor personal and environmental hygiene. Many of the Black people included in this study came from informal settlements with undesirable housing and ablution facilities, which could account for the higher prevalence of GBS in this group, particularly since the majority reported hand basin body washing (which facilitates translocation of GBS) as their practice of personal hygiene. Although the location of the MOU (Midwife Obstetrics Unit) showed a significant association with GBS colonization, race (probably due to the uncategorised persons) did not. Also of note, is that the Mowbray MOU serves both race groups. Personal hygiene, employment, level of education, marital status, and age showed no significant association with GBS colonization.

A prevalence rate similar to this study (16.5%) was reported from Germany (20) and 17.2% reported from the Netherlands (21). Lower GBS prevalence (3.2%) was reported from Argentina (22), Turkey (8.0%) (23), China (8.2%) (24), Mozambique (1.8%) (25), India (2.3%) (26), and Nigeria (10.7%) (27). The higher prevalence rates (25, 30, and 30.9%) reported in the other South African studies [(17–19), respectively], fall within the range reported from other parts of Africa such as 20.9% in Ethiopia (28), 23% in Tanzania (29), and 31.6% in Zimbabwe (30). Studies from Iran (31), Brazil (32), and Switzerland (33) also reported prevalence rates of 22.7, 24, and 21%, respectively.

Variations between different countries could be due to multiple factors including time of sampling (i.e., during pregnancy or at delivery), sampling site (vaginal only vs. vaginal plus anorectal) and detection techniques (enriched selective vs. enriched media and /or PCR) as well as maternal socio-economic and other personal demography (34, 35). In their systematic review and meta-analyses of maternal GBS colonization world-wide, (35) reported an overall prevalence of 15% regardless of sample collection or microbiological techniques used. After adjusting for sampling and microbiological methods, the overall prevalence increased to 18%, thereby demonstrating the risk of underestimation when doing comparative studies.

Previous reports indicated that while GBS were isolated from only rectal specimens in some cases and from vaginal specimens in other cases, swabbing both the vagina and rectum was found to increase the yield substantially compared with sampling either the vagina or the rectum alone (26). Eight women tested positive for GBS in both vaginal and rectal sites in this study and 5 of the 8 women had identical isolates in both sites, with the remaining 3 women colonized with two different serotype combinations. Had we excluded rectal samples from our study, it would have resulted in false negative results in 40% of cases. We observed that the increased recovery from the rectal samples was largely due to the incubation in selective enriched broth media for 24 h prior to plating onto enriched agar. Conversely, a study by (36) compared different cultural methods and reported poor recovery of GBS from rectal swabs using selective broth enrichment. The fact that GBS was sometimes isolated from only one site and not the other shows how imperative it is to sample both anatomical sites (vagina and rectum) when screening for GBS carriage in pregnant women.

Maternal HIV status did not significantly affect GBS colonization, nor did smoking or alcohol consumption in this study. Whether or not HIV positivity contributes to GBS colonization remains inconclusive. A recent study from Nigeria (27) reported an overall prevalence of 10.7% GBS, with increased GBS colonization in HIV-positive (15.5%) compared to HIV-negative (6.0%) mothers, while HIV infection was not considered as a risk factor for GBS colonization in a study from USA (37), although age, ethnicity/race, alcohol, and tobacco showed highly significant relationships. A South African study also reported no maternal risk for increased GBS colonization in HIV+ pregnant women (38) although there have been reports of infants born of HIV+ mothers being at risk for invasive GBS disease (39). Another South African study which examined the effect of maternal HIV infection on immunoglobulin G serotype specific-capsular (1a, 1b, III, and V) antibody along with GBS surface protein maternal antibodies and transplacental transfer to their neonates, revealed that after adjusting for overall and serotype-specific GBS colonization, maternal age and parity, the cord-maternal ratio was significantly lower for serotypes 1a (*p* < 0.0001) and III (*p* = 0.027) in HIV+ compared to HIV– mother-neonate dyads, supporting the hypothesis of an increased risk for neonatal GBS infection with reduced protective antibodies (40).

The above comparison of GBS serotypes detected in HIV+ and HIV– mothers in Soweto and surrounding areas in South Africa (40) showed that serotypes III and Ia were more prevalent in HIV– mothers (40.7 and 59.1%, respectively), than in HIV+ mothers (13.6 and 29.6%, respectively), while in Brazil, serotypes Ib (34.9%) and Ia (25.6%) predominated in HIV+ women (41). Our study showed no significant differences in serotype distribution between the HIV+ and HIV– mothers for serotypes III and V, although serotypes II, IV, and IX were found in HIV- mothers only and not in HIV+ mothers.

The distribution of GBS serotypes not only differs from one country to another but also between provinces within the same country, with changes in prevalence over time (24, 42). A recent global survey reported that serotypes Ia, Ib, II, III, and V account for 98% of GBS serotypes detected, with variations reported for different regions (35). A similar meta-analysis of GBS maternal disease (43), listed serotype Ia as the most prevalent (31%), followed by III (27%), V(19%), Ib(14%), and II(5%). Serotypes 1a and III are reported to account for the majority of invasive cases of GBS (4, 44), while other studies (42, 45) reported serotype V as the most important serotype in invasive disease. In this study, 6 different serotypes of GBS were identified with serotype V predominating, followed by serotype III. Of the 8 women who tested positive for GBS in both vaginal and rectal sites in this study, 5 had identical isolates in both sites, while the remaining 3 were colonized with two different serotype combinations, each of 2 combinations including either serotype III or serotype V (i.e., serotype combinations III/IX, IV/V) and 1 with both III/V. Serotype III GBS strains are considered to account for the majority of infections in neonates worldwide (46), with invasive strains reported in Nairobi (47) and Soweto, South Africa (45). Serotype III was also the most prevalent (29.7%) in another study conducted in Pretoria, South Africa (18), followed by serotypes Ia (25.8%), IV (10.9%), and Ib (8.6%). Serotypes Ib, VI–VIII were not present in our study nor were VI–VIII found in the study of (18). The South African study which examined the effect of maternal HIV infection on immunoglobulin G serotype specific-capsular (1a, 1b, III, and V) antibody along with GBS surface protein maternal antibodies and transplacental transfer to their neonates, revealed that after adjusting for overall and serotype-specific GBS colonization, maternal age and parity, the cord-maternal ratio was significantly lower for serotypes 1a (*p* < 0.0001) and III (*p* = 0.027) in HIV+ compared to HIV– mother-neonate dyads, suggesting an increased risk for neonatal GBS infection with reduced protective antibodies (40).

Three isolates (5%) were non-typeable in the present study, while only 0.7% of isolates were non-typeable in the Pretoria study (18). In the absence of human error, non-typeability might be explained if the isolate has an insertion or a mutation in genes that are essential for capsule expression (48), resulting in a non-encapsulated variant or, if the isolate produces an uncharacterized polysaccharide for which antibodies are not yet available (i.e., a new serotype). The multiplex PCR method of (49) yields good sensitivity and specificity and enables the characterization of all known GBS serotypes, thereby reducing the rate of detection of non-typeable isolates. However, due to financial constraints, we were unable to apply this technique and report on the latex agglutination method instead.

Just as different serotypes predominate in different population groups, so do their antimicrobial profiles. GBS susceptibility has frequently been reported to ampicillin, cefazolin, erythromycin, imipenem, norfloxacin, penicillin, and vancomycin (32). Penicillin G remains the antibiotic of choice for the intrapartum prophylaxis for GBS, with erythromycin and clindamycin used as alternatives for those allergic to penicillin (26). Antibiotic resistance patterns of 57 GBS isolates (from 50 mothers) were tested in this study for their susceptibility to eight different types of antibiotics that have been recommended for the eradication of carriage and treatment of invasive GBS diseases. Results showed that few were resistant to penicillin (5%), erythromycin (2%), and clindamycin (2%) compared to resistance frequencies of 21.1% and 17.2% reported for erythromycin and clindamycin in other studies (50, 51). Although strains resistant to penicillin have been described in previous studies, they remain rare (52, 53). Resistance to clindamycin and erythromycin is on the increase and along with amoxicillin, have not been successful in reducing adverse pregnancy outcomes (54). This resistance has been attributed to specific serotypes.

Serotypes III and V are reported as the most resistant serotypes with resistance to erythromycin, trimethroprim/sulphamethoxazole (TM/SMX), clindamycin and tetracycline reported (32, 55, 56). In the present study, serotype III and V demonstrated resistance to tetracycline, TM/SMX, with susceptibility to most of the other antimicrobials tested, as did Ia, II, and IX.

Strategies to prevent neonatal GBS infection include the elimination of exposure to GBS, chemoprophylaxis and vaccines. Intrapartum chemoprophylaxis has greatly reduced the risk of neonatal infection in women who carry GBS (5), but despite these measures, GBS remains a leading cause of infectious neonatal morbidity worldwide. The Centers for Disease Control recommend that only mothers with a history of previous PTD should be treated with antibiotics if found to have vaginosis or vaginitis, since previous PTD is a powerful indicator of risk for PTD (8).

All the GBS strains isolated in the present study were resistant to fusidic acid, while others demonstrated resistance to TM/SMX (98%), tetracycline (98%), and gentamicin (93%). Resistance of GBS to tetracycline in our study population was similar to the results observed in other studies (32, 57). In our study, susceptibility to sulphamethoxazole was found in only three women, two of whom were HIV positive. In Brazil, resistance to tetracycline, clindamycin, and trimethroprim/sulphamethoxazole has been reported in asymptomatic GBS carriers (32).

A strength of this study is that to our knowledge it is the first to report on the antimicrobial resistance of GBS serotypes in the Western Cape as well as serotype differences in HIV+ vs. HIV– mothers. Limitations of the study include the relatively small sample number, reluctance of some participants to reveal their racial or HIV status, low income settings biased toward urban areas thereby eliminating a comparison with rural or advantaged socio-economic groups, and failure to follow up on neonates born of these mothers.

GBS culture is labor intensive with the possibility of mixed cultures resulting in the overgrowth of competitive species and incubation time. Molecular diagnostic techniques such as PCR have the advantage of rapidly identifying bacteria which are difficult to grow and identifying newly emerging strains of bacteria. The benefit of rapid reporting of bacteria in pregnant women can significantly impact their pregnancy outcomes and reduce mortality rate, while allowing for a simpler and more efficient prevention programme which is speedy, sensitive, and precise. Thus, PCR offers a good alternative to culture in the absence of susceptibility testing, with 100% sensitivity and specificity reported in the present study when compared with culture.

## Conclusion

In conclusion, the results of our study differ considerably from studies in other regions of South Africa as well as in other parts of the world, with differences in serotype distribution and antimicrobial susceptibility possibly serving as a basis for GBS guidelines for South Africa. Our results stress the importance of epidemiological updates, particularly for those with penicillin allergies. More studies are needed to accurately estimate the global burden of GBS especially in developing countries (30). Improved knowledge of the prevalence of GBS in pregnant women would lead to a greater understanding of EOD and LOD potential.

## Author Contributions

CA designed the study, supervised the laboratory work and wrote the final paper. EK was responsible for the data collection, *in vitro* laboratory investigations and preparation of the first draft of the manuscript. Both authors read and approved the final manuscript.

### Conflict of Interest Statement

The authors declare that the research was conducted in the absence of any commercial or financial relationships that could be construed as a potential conflict of interest.

## Abbreviations

EOD: Early Onset Disease
GBS: Group B *Streptococcus*
LOD: late onset disease.
